# Evaluation of the immature platelet fraction as a predictive marker of bone marrow regeneration after hematopoietic stem cell transplantation

**DOI:** 10.1111/ijlh.14358

**Published:** 2024-09-04

**Authors:** Kélian Steibel, Magalie Joris, Valentin Clichet, Amandine Charbonnier, Judith Desoutter, Jean‐Pierre Marolleau, Loïc Garçon, Thomas Boyer

**Affiliations:** ^1^ Laboratory of Hematology Amiens‐Picardie University Hospital Amiens France; ^2^ Department of Hematology Amiens‐Picardie University Hospital Amiens France; ^3^ Laboratory of Hematology Saint Louis hospital Paris France; ^4^ Laboratory of Histocompatibility Amiens‐Picardie University Hospital Amiens France; ^5^ UR 4666 HEMATIM Université Picardie Jules Verne Amiens France

**Keywords:** hematopoietic stem cell transplantation, immature platelet fraction, neutrophils recovery, platelet recovery

## Abstract

**Introduction:**

Hematopoietic stem cell transplantation (HCST) is a widely used therapy in the management of hematological malignancies, leading to cytopenias that require transient transfusions. Platelet recovery (PR) following HSCT is assessed by monitoring platelet count (PC). Immature platelet fraction (IPF) is a research parameter offered by Sysmex® on XN series analyzers, enabling rapid diagnostic orientation in the event of thrombocytopenia. It has also been described as a predictive factor for PR after chemotherapy or HSCT, and thresholds have been proposed.

**Methods:**

The objective of this study was to assess the predictive capability of IPF for PR in a prospective cohort of patients undergoing HSCT and to evaluate its utility in guiding platelet transfusion decision.

**Results:**

An optimized A‐IPF (absolute number of IPF) threshold of 2.5 × 10^9^/L was predictive of a PC greater than 50 × 10^9^/L at day 30 with a sensitivity of 78.9%, specificity of 78.6%, positive predictive value (PPV) of 83.3% and negative predictive value (NPV) of 73.3%. We were able to distinguish patients recovering PC before day 15 with an earlier %IPF peak, greater IPF recovery kinetics and faster neutrophil recovery.

**Conclusion:**

A‐IPF shows promise as a predictor of PR following HSCT. A multicenter study could help confirm both A‐IPF and %IPF (IPF) clinical utility before it is made available to clinicians.

## 1 INTRODUCTION

In 1969, Ingram and Coopersmith^1^ were the first to discover immature platelets (IP), then called reticulated platelets because of their analogy with reticulocytes. Afterwards, Ault and Knowles^2^ demonstrated that IP are the youngest platelets, newly released into the general circulation, with a lifespan of 24 h to 1.4 days.^2^, ^3^ The concentration of these IP is thought to be proportional to the density of megakaryocytes,^4^ therefore reflecting megakaryopoiesis.^4^, ^5^ The quantification of IP has evolved from manual methods,^5^ such as thiazol orange (TO) labeling proposed by Kienast and Schmitz, to automated techniques. Initially, Toa Medical Electronics introduced the R‐3000,^6^ featuring auramine O RNA labeling for platelet nucleic acids. Presently, the Sysmex® XE^7^ and XN^8^ series offer automated systems equipped with research parameters like the immature platelet fraction (IPF), a reflect of IP, providing improved diagnostic capabilities. Particularly, on the XN series, platelet RNA is labeled with oxazine^9^ on a specific fluorescent channel (PLT‐F channel), and an algorithm is subsequently used to differentiate mature platelets from IP. The %IPF is calculated as the proportion of large platelets with high RNA content relative to the total platelet count (PC) and the absolute number (A‐IPF) can also be determined. The potential of IP to help in the etiological diagnosis of thrombocytopenia has been a subject of exploration since the availability of this parameter on new generation analyzers.^5^ Various thresholds have been proposed^10^, ^11^, ^12^ to differentiate thrombocytopenia of peripheral origin using %IPF in order to avoid bone marrow (BM) aspiration^13^ for patients with suspected idiopathic thrombocytopenic purpura (ITP). For example, a threshold of 13% IPF^14^ has been proposed with a sensitivity (Se) and positive predictive value (PPV) of 100%.

An increase of %IPF has been observed in patients during the recovery phase of thrombocytopenia,^5^ including patients undergoing hematopoietic stem cell transplantation (HSCT), occurring a few days before the rise in PC.^6^ The field of application of IPF could therefore be extended to the prediction of platelet recovery (PR) in patients with hematological diseases, after a course of chemotherapy with or without HSCT. Usually, during the initial phase after HSCT, IPF levels are low,^15^ corresponding to the nadir, before significantly increasing over the subsequent 10–26 days, preceding PR. In the Sysmex® XN series, a cutoff of 5.3%^16^ demonstrated the highest area under the curve (AUC) for predicting PR within 2 days in patients undergoing autologous stem cell transplantation, outperforming results from the XE series. A recent study by an Australian team evaluated the capacity of IPF on XN series to predict PR on a cohort of allo‐HSCT patients,^17^ using the definition of PR with the recommendations of the Center for International Blood & Marrow Transplant Research (CIBMTR).^18^ Consequently, the mean IPF between day 11 and day 15 emerged as the most sensitive predictor for PC exceeding 50 × 10^9^/L at day 30. Some authors^19^, ^25^ even assumed for some patients in their cohort that platelet transfusions would be unnecessary, based on %IPF. In this prospective study, we assessed the predictive capability of IPF for PR in patients undergoing HSCT and its utility in guiding platelet transfusion decision.

## 2 MATERIALS AND METHODS

### 2.1 Study design and patients

This prospective, non‐interventional study was carried out between September 2022 and April 2023. The selected patients were adults monitored at Amiens University Hospital for hematological malignancies, and all underwent HSCT. Our study was based on the collection of EDTA whole blood samples (biological waste) from patients during their monitoring at the hospital. A total of 33 patients were included. Patients were classified by PR group with the early recovery group with PR within 15 days of transplantation, the late recovery group with a PR between day 16 and day 30, and finally the not recovering group. The definitions of platelet and neutrophil recoveries used here are in accordance with CIBMTR^18^ recommendations. PR is defined as the first day on three different consecutive days with a PC greater than 20 × 10^9^/L, without platelet transfusion. Neutrophil recovery (NR) is defined as the first day on three different consecutive days with an absolute neutrophil count (ANC) greater than 0.5 × 10^9^/L.

### 2.2 Data collection

Patients were followed for 30 days (sometimes up to 33 days) after transplantation. Complete blood count (CBC) parameters such as hemoglobin concentration (g/dL), ANC (10^9^/L), PC (10^9^/L), mean platelet volume (MPV, fL) as well as IPF (% and 10^9^/L) were collected daily. CBC parameters were obtained from whole blood collected in EDTA‐K2 tube (Vacutainer, Beckton Dickinson®) after analysis on the XN‐1000 (Sysmex®, Kobe, Japan). Clinical data, such as the graft source (peripheral blood [PBSC] or bone marrow [BMSC] stem cells), quantity of grafted cells (CD33+, CD3+, and total nucleated cells [TNC] per kg), histocompatibility (pheno‐, haplo‐, and geno‐identical), type of conditioning regimen (reduced intensity conditioning [RIC], myeloablative conditioning [MAC], and myeloablative‐reduced toxicity conditioning [MA‐RTC]), presence of acute graft versus host disease (GVHD), post‐transplant transfusions of red blood cell (RBC), and platelet concentrates, were collected.

### 2.3 Statistical analysis

Descriptive analysis is detailed as mean ± standard deviation (SD) or median with range. Platelet and neutrophil recovery times were analyzed by Kaplan–Meier method. Maximum A‐IPF and %IPF were compared between groups by post hoc test with Bonferroni correction. The delays between the occurrence of the first peak and the maximum value %IPF were compared between groups by Krukal‐Wallis test. The correlation between PR and graft quality (source and cellularity) or the nature of conditioning was tested by COX regression or Pearson correlation. The mean slopes of the increase in %IPF and A‐IPF (from −7 to +7 days around PR as day 0) between the early and late groups were compared using a Wald test. Predictive power of A‐IPF and %IPF was assessed on receiver operating characteristic (ROC) curves by comparing AUCs. For the different thresholds proposed, sensitivity, specificity, PPV, and negative predictive value (NPV) were calculated. All tests were two‐sided, statistical significance was defined as a *p*‐value <0.05, and statistical analyses were performed with R software 4.0.5 and Python 3.1.

## 3 RESULTS

### 3.1 General cohort

A total of 33 patients were included in our study (sex ratio = 1.06), with a median age at transplantation of 56 years old (20–71). The characteristics are summarized in Table 1. Indications for allogeneic transplantation were distributed as follows: de novo AML (*n* = 11), secondary AML (*n* = 3), B‐cell acute lymphoblastic leukemia (B‐ALL) (*n* = 6), T‐cell acute lymphoblastic leukemia (T‐ALL) (*n* = 1), myelodysplastic syndrome (MDS) (*n* = 4), MDS/myeloproliferative syndrome (MPN) (*n* = 2), non‐hodgkin lymphoma (NHL)‐T (*n* = 1), idiopathic aplastic anemia (*n* = 1), primitive myelofibrosis (*n* = 1), and secondary myelofibrosis (*n* = 3). Seventy‐six percentage of the patients received PBSC allografts, compared with 24% of BMSC. No cord blood stem cell transplants were performed. Fifty‐eight percentage of HSCT were pheno‐identical, 27% haplo‐identical, and 15% geno‐identical. Seventy‐nine percentage of the patients had a RIC conditioning, 3% a MA‐RTC conditioning, and a 18% MAC conditioning. A total of 25 patients (75.8%) fulfilled the definition of PR within 30 days. Two patients died during collection. Only one patient showed no evidence of graft uptake at the end of the follow‐up, with neutropenia and severe thrombocytopenia at day 30. This patient received a pheno‐identical 10/10 PBSC allograft and was diagnosed with acute GVHD.

**TABLE 1:** General cohort data.

Cohort	*n* = 33
Sex	
Men	17 (52%)
Women	16 (48%)
Sex ratio	1.06
Age (years)	
Mean	53
Median	56
Range	[20–71]
Diagnoses	
de novo AML	11
Secondary AML	3
B‐ALL	6
T‐ALL	1
MDS	4
MDS/MPN	2
NHL‐T	1
Idiopathic aplastic anemia	1
Primitive myelofibrosis	1
Secondary myelofibrosis	3
Matching	
Haplo‐identical	27%
Geno‐identical	15%
Pheno‐identical	58%
Graft source	
PBSC	76%
BMSC	24%
Conditioning	
MAC	18%
MA‐RTC	3%
RIC	79%

*Note*: This table shows the various data for the general cohort.

Abbreviations: AML, acute myeloid leukemia; ALL, acute lymphoblastic leukemia; MDS, myelodysplastic syndrome; MPN, myeloproliferative neoplasm; NHL, non‐Hodgkin lymphoma; PBSC, peripheral blood stem cells; BMSC, bone marrow stem cells; MAC, myeloablative conditioning; MA‐RTC, myeloablative‐reduced toxicity conditioning; RIC, reduced intensity conditioning.

Median NR was 17 days (10–27). Mean hemoglobin concentration in the whole cohort remained stable throughout the collection period, between 8 and 10 g/dL (9.2 ± 1.4 g/dL). A total of 200 RBC and 296 platelet concentrates were consumed over the course of the study. While hemoglobin did not exhibit a significant association (Hazard Ratio [HR] = 1.28, 95% confidence interval [CI]: 0.86–1.89, *p*‐value = 0.2), both %IPF and A‐IPF demonstrated strong positive associations with PR in the univariate analysis (respectively HR = 1.10, 95% CI: 1.03–1.18, *p*‐value = 0.006 and HR = 1.60, 95% CI: 1.30–1.98, *p*‐value <0.001). Transitioning to the time‐dependent multivariate analysis, the findings corroborated the significant association observed for A‐IPF. A‐IPF maintained a positive association with PR (HR = 2.78, *p*‐value = 0.009), suggesting a substantial impact on recovery. While trends were observed for %IPF and hemoglobin concentration, their associations did not reach statistical significance (respectively HR = 1.14, CI: 0.98–1.32, *p*‐value = 0.081; and HR = 1.31, CI: 0.85–2.02, *p*‐value = 0.229).

### 3.2 Platelet recovery groups

#### 3.2.1 Early recovery group

**FIGURE 1:**
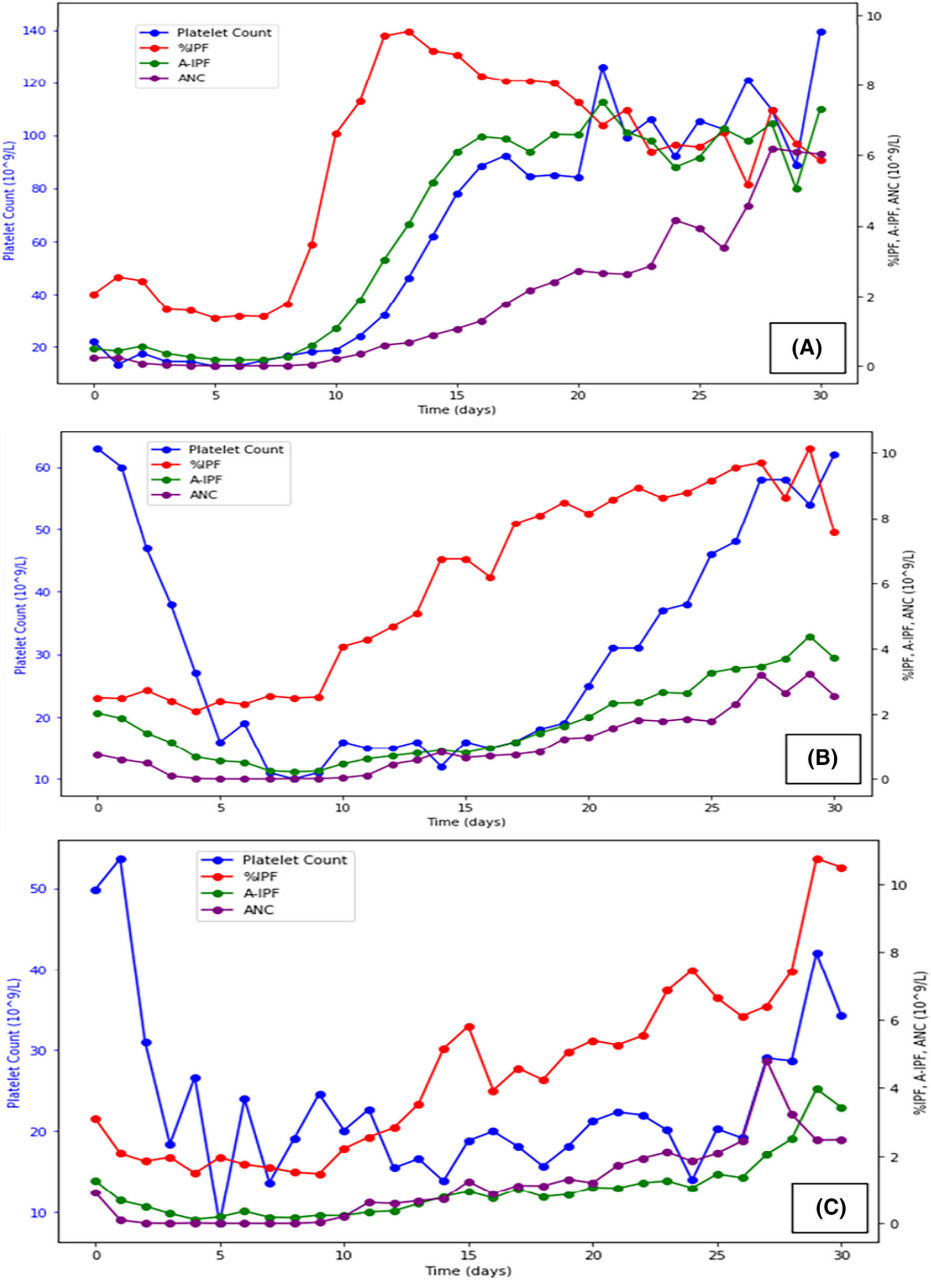
Kinetics of the means PC, %IPF, A‐IPF, and ANC over time, post‐allograft, according to the recovery group. Evolution of the mean platelet count (PC, 10^9^/L; blue curves), immature platelet fraction (IPF: %IPF [percentage, red curves] and A‐IPF [absolute count, 10^9^/L, green curves]), and absolute neutrophil count (ANC, 10^9^/L: purple curves) over the 30 days following allograft. Chart 1 (A): early recovery group; Chart 2 (B): late recovery group; Chart 3 (C): not reached recovery group. A pattern of rapid PR stands out for the early group, with an increase initially in %IPF, then in A‐IPF and PC, shortly before NR. The parameter that increases first for the late recovery group is %IPF. The increase in other markers is less marked than for the early group. For the group of patients who have not recovered, the various parameters evolve in an anarchic way.

For the 16 patients (48.5% of the cohort) included in the early group, PR was achieved with a median of 12 days (9–15). All the results are summarized in Table 2. The mean of the maxima %IPF was 11.8 ± 3.7%, and they were achieved with a median of 12 days (10–28), on average 1.1 ± 4.5 days after PR. This pattern was identifiable on the curves of all patients in this group with a peak in %IPF, visible on the kinetics of the mean %IPF (Figure 1A). The mean A‐IPF for this group increased from day 8 (0.3 ± 0.2 × 10^9^/L) and steadily raised until the end of the collection to reach an average value of 6.4 ± 3.6 × 10^9^/L. The mean value of the maxima A‐IPF was 8.8 ± 3.5 × 10^9^/L, they were achieved with a median of 21.5 days (15–28).

**TABLE 2:** Characteristics of platelet recovery groups.

	Early group	Late group	Not reached group
*n*	16	9	8
Age (years): median, range	61.5 [37–71]	53 [30–67]	40 [20–68]
Sex ratio	1	1.25	1
%IPF (%)			
Mean ± SD	5.4 ± 4.1	5.9 ± 5.5	4.3 ± 3.8
Range	[0–19.6]	[0–24.2]	[0.2‐17.7]
Maximum: mean ± SD	11.8 ± 3.7	13.3 ± 6.4	9.9 ± 4.8
Day of maximum, range	12 [10–28]	22 [17–30]	26.5 [14–31]
95p	13.3	18.2	13.3
99p	16.3	22.0	15.8
1st %IPF peak			
Day: median, range	12 [10–15]	18 [14–28]	23 [13–31]
Value (%): mean, range	11.5 [5.1‐19.6]	12,6 [5.1‐24.1]	9,3 [4.8‐17.7]
A‐IPF (10^9^/L)			
Mean ± SD	3.3 ± 3.4	1.6 ± 1.7	0.9 ± 1.2
Range	[0–18.9]	[0–7.2]	[0–7]
Maximum: mean ± SD	8.8 ± 3.5	4.7 ± 1.6	2.4 ± 2.1
Day of maximum, range	21.5 [15–28]	29 [21–30]	23 [13–30]
95p	9.7	5.2	3.1
99p	13.2	6.4	6.1
PR (days): median, range	12 [9–15]	22 [16–28]	NA
NR (days): median, range	15 [10–19]	19 [11–27]	18 [10–26]
PC d25‐d30 (10^9^/L): mean ± SD	111.2 ± 50.1	54.1 ± 44.9	27.3 ± 18

Abbreviations: SD, standard deviation; 95p, 95th percentile; 99p, 99th percentile; IPF, immature platelet fraction (in percentage [%IPF] or absolute count [A‐IPF, 10^9^/L]); PR, platelet recovery; NR, neutrophil recovery; PC, platelet count; d, day.

#### 3.2.2 Late recovery group

For the nine patients (27.3% of general cohort) who belonged to the late group, PR was achieved with a median of 22 days [16–28]. The mean of the maxima %IPF was 13.3 ± 6.4% and this was observed with a median of 22 days (17–30). An increase in mean %IPF was visible from day 9 (2.5 ± 2.5%) and was constant until day 27 (9.7 ± 5.7%) (Figure 1B). The %IPF peak pattern was not identifiable on the kinetics of all patients, nor on the group mean %IPF curve (Figure 1B). Mean A‐IPF increased from day 9 (0.2 ± 0.2 × 10^9^/L) and was constant up to the maximum. The median A‐IPF maxima for this group was day 29 (21–30). The mean value of these maxima was 4.7 ± 1.6 × 10^9^/L. In general, we observed increasing dissociated kinetics between A‐IPF and PC; this might explain the absence of a %IPF peak on the mean curve for this group.

#### 3.2.3 Not reached recovery group

This group included eight patients (24.2% of general cohort). Two patients died during the data collection. The increase in mean %IPF for this group was irregular from day 9 (1.5 ± 0.5%) to the end of the collection period (Figure 1C), with a transfusion requirement still present at this stage. The mean of the maximum %IPF was 9.9 ± 4.8%, and this maximum value was reached with a median of 26.5 days (14–31). The increase in mean A‐IPF was comparable to those of the late group, with lower values (mean maximum A‐IPF = 2.4 ± 2.1 × 10^9^/L) and obtained with a median of 23 days (13–30). Inter‐individual variability was substantial for all the biological parameters observed in this group.

#### 3.2.4 Neutrophil recovery

The median NR was 15 days (10–19) for the early group, compared to 19 days (11–27) for the late group and 18 days (10–26) for the not reached group. Only one patient did not recover a normal ANC at the end of the collection.

PR was closely related to NR, so classification of patients according to PR highlighted an earlier NR for patients with early PR compared to the late group (*p*‐value = 0.012) and early PR was positively associated with faster NR (*p*‐value = 0.01).

##### Platelet recovery kinetics and IPF thresholds

The slopes of %IPF and A‐IPF recoveries were significantly different between the early and late groups (*p*‐values of 0.025 and <0.001, respectively). The kinetics of the increase in IPF were greater in the early PR group. Maxima of %IPF and A‐IPF were significantly different (*p*‐value = 0.008 and <0.001, respectively) and the A‐IPF peak was the most powerful tool to discriminate between early from the late group (*p*‐value = 0.004 and 0.001, respectively). The %IPF peaks of the early group were significantly different from the not reached group (*p* = 0.013) but not from the late group (*p* = 0.116). Furthermore, there was no linear correlation between the maximum %IPF and the PR time for the 25 patients who recovered (*R* ^2^ = 0.072).

For the not reached group, the first %IPF peak occurred at a median of 23 days (13–31), compared with 12 days (10–15) for the early group and 18 days (14–28) for the late group (Table 2). These first %IPF peaks occurred earlier in the recovery phase for the early group than for the late group (*p*‐value = 0.04) and the non‐recovered group (*p*‐value <0.001).

**FIGURE 2:**
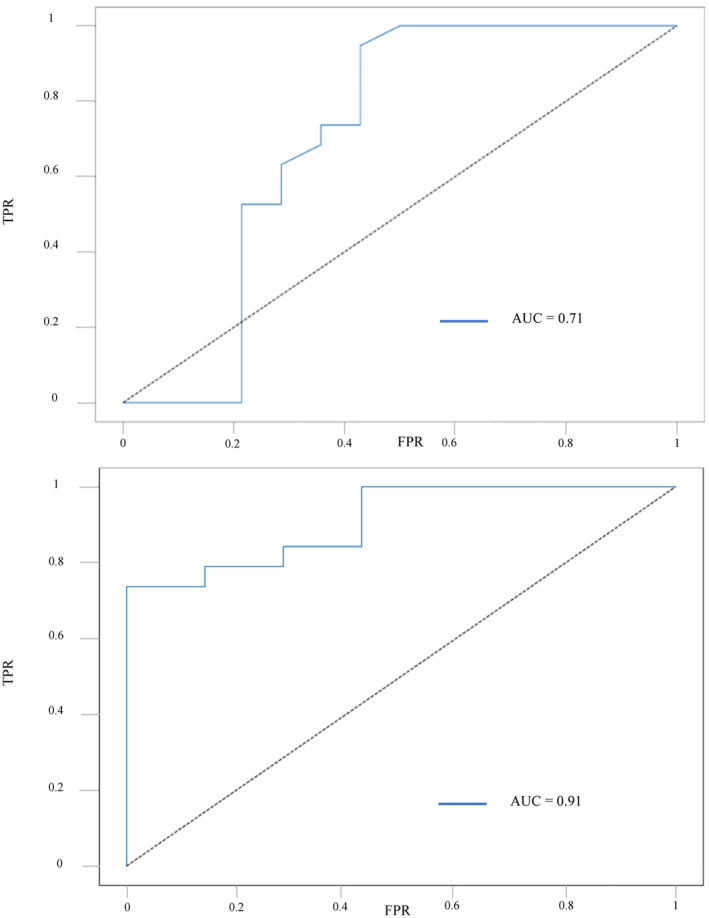
%IPF and A‐IPF ROC curves. On top: receiver operating characteristic (ROC) curve of percentage immature platelet fraction (%IPF) on predicting a platelet count (PC) above 50 × 10^9^/L at day 30 post‐graft (area under curve (AUC) = 0.71). At the bottom: ROC curve of absolute immature platelet fraction (A‐IPF) on predicting a PC above 50 × 10^9^/L at day 30 post‐graft (AUC = 0.91). A‐IPF shows a better AUC than %IPF.

The ROC curve for %IPF had an AUC of 0.71 (Figure 2). Several %IPF thresholds were tested to predict PC greater than 50 × 10^9^/L at day 30. The optimized sensitivity threshold (100%) was 4%, with a specificity of 50%, a PPV of 73%, and an NPV of 100%. The optimized threshold for specificity (79%) was 10%, with a sensitivity of 53%, a PPV of 77%, and an NPV of 55%. The %IPF threshold of 2% between day 11 and day 15 showed excellent sensitivity (100%) with, however, a specificity of 29% (10 false positives), the PPV was 65.5% and the NPV was 100%. The AUC of A‐IPF was higher than that of %IPF (0.91, Figure 2). The optimized threshold for specificity (100%) was 4 × 10^9^/L, with a sensitivity of 57.9%, a PPV of 100% and an NPV of 73%. An optimized threshold of 2.5 × 10^9^/L had a sensitivity of 78.9%, a specificity of 78.6%, a PPV of 83.3%, and a NPV of 73.3%.

#### 3.2.5 Conditioning

Conditioning was not significantly associated with a faster PR (*p*‐value = 0.195) and only a trend was observed with RIC with a median PR of 15 days (9–28), compared with 27 days (16–27) for patients receiving MAC (*p*‐value = 0.09).

#### 3.2.6 Graft quality

##### Graft source and HLA compatibility

Histocompatibility was not significantly correlated with PR (*p*‐value = 0.643) but PBSCs were significantly more associated with an early recovery (*p*‐value = 0.022).

##### Graft cellularity

We lacked data at this level, for graft cell counts (2 patients) and for TNCs (8 patients). No association was found between these thresholds and the time to PR, except for CD34+ cells (*p*‐value = 0.005). Patients transplanted with TNC levels below 8 × 10^8^/kg were more represented in the late group. A total of 14 of the 20 patients with levels above this threshold recovered their PC within the first 15 days.

#### 3.2.7 Platelet transfusion needs

A total of 296 platelet concentrates were used during the collection period. A total of 23 transfusions were administered above the A‐IPF threshold: 3 transfusions (4 concentrates) for the early group, 8 (9 concentrates) for the late group, and 12 (15 concentrates) for the not reached group. Platelet concentrates consumption differed significantly between the three groups (overall *p*‐value <0.001).

**FIGURE 3:**
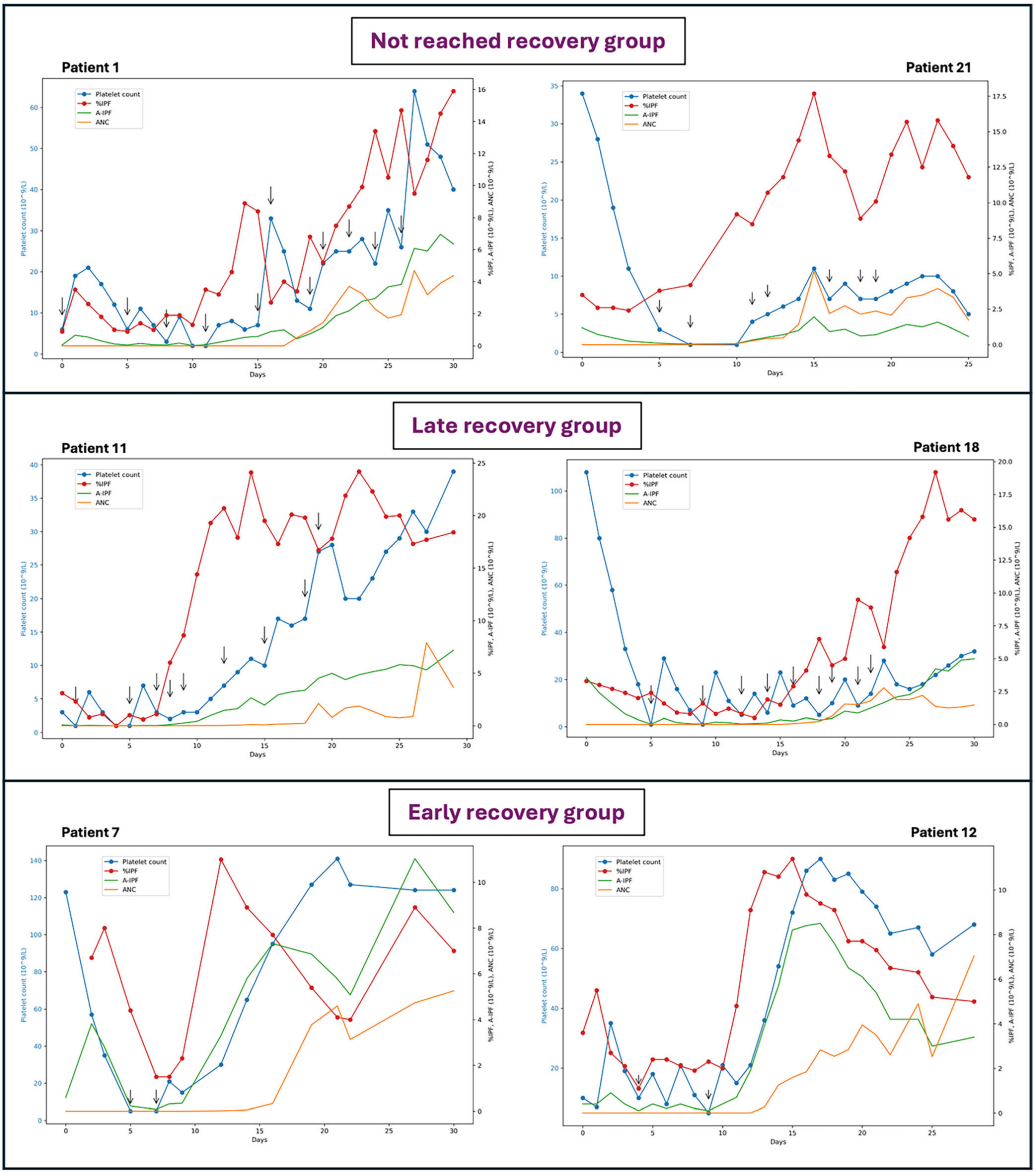
Kinetics of hematological parameters and platelet transfusion of various patients. These charts represent the kinetics of the different hematological parameters: immature platelet fraction (IPF: %IPF in red, A‐IPF [109/L] in green), absolute neutrophil count (ANC, 109/L) in orange, platelet count (PC, 109/L) in blue, after hematopoietic stem cell transplant (HSCT) as well as the transfusions that took place (black arrows). Patients 1 and 21 belonged to the non‐recovery group, patients 11 and 18 to the late recovery group and patients 7 and 12 to the early recovery group. Patient 21 is an example of a not reached recovery and died at day 26 post HSCT from acute respiratory distress (ARD).

For the not reached group, requirements were practically constant. The graphs for patients 1 and 21 show a high platelet turnover. The %IPF increased in a sawtooth pattern according to the transfusions. PC also increased, but to a variable degree; this growth was supported by numerous platelet transfusions (e.g., three concentrates at day 26 for patient 1). The %IPF and/or A‐IPF could reach high values, but this did not translate into a sustained rise in PC. In the late recovery group, platelet transfusions were regular, as shown in the graphs for patients 11 and 18 (Figure 3). For the early recovery group, the need for transfusion was essentially present at the PC nadir, i.e. in the first few days after HSCT, with very few transfusions in this group.

Patients 11 (late recovery group) and 16 (early recovery group) received a final platelet concentrate at day 19 (Figure 3) and day 13 (Figure S1) respectively. On these days, the A‐IPF threshold of 2.5 × 10^9^/L and the first %IPF peak had been reached. These transfusions could potentially have been avoided if these biological parameters had been considered.

## 4 DISCUSSION

Automated analyzers are now an integral part of routine medical laboratories, particularly in the field of cellular hematology.^8^ Thanks to advances in analytical techniques, new parameters, “research parameters,” are now available in the pathologist's work environment. Those available on XN analyzers have already been studied to establish their relevance to the clinician. Some parameters are included in nephrology guidelines (EBPG and NKF‐KDOQI^20^). Despite advances in research, the standardization of IP remains a challenge, as well as their routine use. Nevertheless, IPF has already been associated with numerous clinical uses, such as the etiological diagnosis of thrombocytopenia,^14^ monitoring the efficacy of antiplatelet drugs,^21^ and predicting PR.^15^, ^17^, ^22^ However, previous studies suffered either from a lack of a homogeneous cohort, or from the absence of validation of the proposed thresholds by multicenter prospective studies.

In our study, we were able to highlight the importance of IPF in predicting PR after allo‐HSCT. Classification of patients in our cohort into PR groups revealed a common %IPF pattern (first peak) for most patients who recovered within 15 days of allo‐HSCT, a steeper %IPF and A‐IPF recovery curve underlining the higher activity of megakaryopoiesis for this group, less transfusion dependency but also earlier neutrophil recovery. A‐IPF appears to be a more relevant marker, as it is not dependent on platelet transfusion, unlike %IPF, as can be seen in the graph of hematological parameters for patient 1 (Figure 3). In addition, it is more predictive of a PR greater than 50 × 10^9^/L at day 30 post HSCT.

However, the clinical benefit was only marginally noticeable in the early recovery group. Indeed, 9 platelet concentrates out of 73 (i.e., 12.3%) were administered in the early recovery group, after the A‐IPF threshold mentioned in our study and after the %IPF peak. The example of patient 16 (Figure S1) shows that the last transfusion could have been avoided based on the biological data, but a catheter hematoma prompted the decision. These transfusion decisions are ultimately at the clinician's discretion and cannot be ruled out solely on biological grounds. For the late recovery group, this concerned 7 transfusions out of 72 (i.e., 9.7%). Although transfusion is not clinically avoidable, IPF could enable clinicians to reduce the amount of concentrates used^23^ per transfusion.

On the other hand, the behavior of IPF in the late recovery and non‐recovery groups was interesting. The increase in %IPF and A‐IPF suggested a higher turnover in patients not recovering a normal PC. This profile may correspond to transplant associated–thrombotic microangiopathy (TA‐TMA) or disseminated intravascular coagulation (DIC), as suggested by Sakuragi et al.^22^ for some patients in their cohort. These results not only point to the predictive power of IPF in BM regeneration after HSCT, but also to a better understanding of the physiology of the recovery in allograft patients. A larger cohort of patients with late or no platelet recovery would be relevant to distinguish the different kinetic profiles of IPF according to clinical situations.

IPF could be made available to clinicians in the same way as other CBC parameters. Furthermore, a regeneration prediction score could be proposed to the clinician based on graft quality, stage of disease at the time of HSCT, histocompatibility and IPF. However, a reference interval and predictive thresholds need to be defined or verified by multicenter studies and accepted by the medical community. Reference intervals have already been proposed by some authors, but with some differences.^14^, ^24^, ^25^, ^26^, ^27^ The causes of inter‐individual and intra‐individual variability need to be clearly defined. Some of these have already been identified, such as platelet transfusions^28^ and the presence of an MPN.^29^ Thresholds have also been proposed, mainly for %IPF. van der Linden et al.,^16^ and Sakuragi et al.,^22^ proposed similar thresholds (5.3% and 5.8%, respectively, for autologous and allogeneic grafts) on XN automats. Grabek et al.^17^ proposed a threshold of 2% between day 11 and day 15 as being predictive of significant regeneration at day 30. In our study, we propose an A‐IPF threshold of 2.5 × 10^9^/L so the message is close, but the methods used to detect the thresholds differ, which is another argument in favor of a multicenter study based on the same standards. It would be also particularly interesting to study the role of IPF according to pre‐transplant conditioning, since it is a factor influencing platelet reconstitution and an additional argument for significantly increasing the number of patients. Given the small number of patients collected, this study lacks statistical power but could give rise to larger‐scale coordinated work involving other hospitals with hematology transplant departments.

## AUTHOR CONTRIBUTIONS

TB and KS designed the research study. TB, KS, VC, JD and LG collected and analyzed the data. MJ, AC, and JPM managed patients and provided clinical data. TB, KS, and VC wrote the paper, which was approved by all the authors.

## CONFLICT OF INTEREST STATEMENT

The authors declare no conflicts of interest.

## Supporting information


**Data S1.** Supporting Information.

## Data Availability

The data that support the findings of this study are available from the corresponding author upon reasonable request.
