# Supplementary material for: Evaluation of the immature platelet fraction as a predictive marker of bone marrow regeneration after hematopoietic stem cell transplantation
Source: Int J Lab Hematol. 2024 Sep 4;47(1):41–50. doi: 10.1111/ijlh.14358 (PMC11725552; doi:10.1111/ijlh.14358)
Supplement: Supplementary file 1 — Data S1. Supporting Information. [file IJLH-47-41-s001.docx]

# Supplements


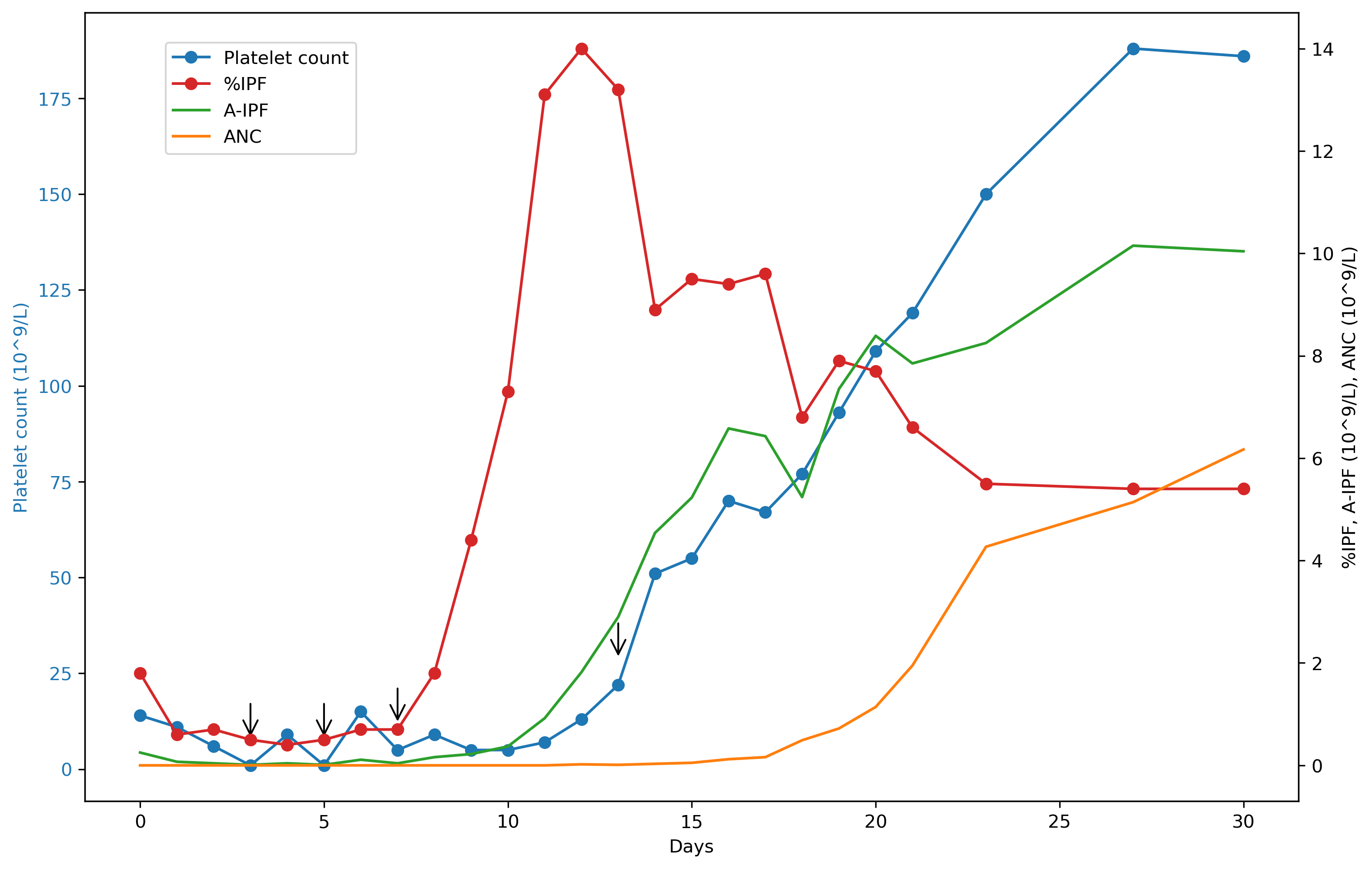


Figure S1 - Kinetics of hematological parameters and platelet transfusion, patient 16

Arrow: Platelet concentrates transfusion (day 3, twice day 5, day 7 and day 13); red curve: percentage Immature Platelet Fraction (%IPF); green curve: Absolute Immature Platelet Fraction (A-IPF, 10^9^/L); blue curve: Platelet Count (PC, 10^9^/L); purple curve: Absolute Neutrophil Count (ANC, 10^9^/L).
